# Resistive switching behavior in Lu_2_O_3_ thin film for advanced flexible memory applications

**DOI:** 10.1186/1556-276X-9-3

**Published:** 2014-01-03

**Authors:** Somnath Mondal, Jim-Long Her, Keiichi Koyama, Tung-Ming Pan

**Affiliations:** 1 Department of Applied Chemistry, National Chi Nan University, Nantou 545, Taiwan; 2 Department of Electronics Engineering, Chang Gung University, Taoyuan 333, Taiwan; 3 Division of Natural Science, Center for General Education, Chang Gung University, Taoyuan 333, Taiwan; 4 Graduate School of Science and Engineering, Kagoshima University, Kagoshima 890-0065, Japan

**Keywords:** Resistive switching, Space-charge-limited-current, Flexible, Nonvolatile memory, Lu_2_O_3_

## Abstract

In this article, the resistive switching (RS) behaviors in Lu_2_O_3_ thin film for advanced flexible nonvolatile memory applications are investigated. Amorphous Lu_2_O_3_ thin films with a thickness of 20 nm were deposited at room temperature by radio-frequency magnetron sputtering on flexible polyethylene terephthalate substrate. The structural and morphological changes of the Lu_2_O_3_ thin film were characterized by x-ray diffraction, atomic force microscopy, and x-ray photoelectron spectroscopy analyses. The Ru/Lu_2_O_3_/ITO flexible memory device shows promising RS behavior with low-voltage operation and small distribution of switching parameters. The dominant switching current conduction mechanism in the Lu_2_O_3_ thin film was determined as bulk-controlled space-charge-limited-current with activation energy of traps of 0.33 eV. The oxygen vacancies assisted filament conduction model was described for RS behavior in Lu_2_O_3_ thin film. The memory reliability characteristics of switching endurance, data retention, good flexibility, and mechanical endurance show promising applications in future advanced memory.

## Background

Resistive switching (RS) behavior, which utilizes the resistance change effect of oxide material, has attracted considerable attention and been widely investigated due to its potential application in future nonvolatile memory (NVM) devices [1]. Several metal oxide materials including NiO [2], TiO_2_[3], Cu_*x*_O [4], and Al_2_O_3_[5] have been studied for resistive random access memory (ReRAM) applications. On the other hand, the flexible electronics are an emerging class of devices in an intriguing technological paradigm. The demand for flexible electronics is revived because of their inherit merits of low cost, light weight, excellent portability, and user-friendly interfaces over conventional rigid silicon technology [6]. Despite these advantages, there is very little in the works about the flexible and NVM devices because of the difficulty to satisfy the dual requirements of memory element. A major challenge for flexible electronics is the lack of good performance NVM devices fabricated at low temperature [7,8]. The ReRAM shows promising memory performance on plastic flexible substrate when processed at low temperature, but the degradation behavior due to excessive mechanical and electrical stress, large switching power, and distribution is the basic limitation for high-density electronic applications [9-12]. It is expected that an achievement of such flexible- and nonvolatile-type memory device will be the next step toward the realization of flexible electronic systems. Recently, flexible resistive memories have been reported in various oxides including graphene oxide (GO) [13], HfO_2_[14], NiO [15], and single-component polymer [16] thin films. However, the huge dispersion in switching parameters, deprived reliability, and poor understanding of the RS behavior are some of the fundamental issues which hinder its application for high-density flexible electronics.

It is well articulate that the amorphous high-κ gate dielectrics, which have already been established to be promising for semiconductor transistor technologies, can be good alternative for ReRAM applications as long as such these materials can perform good RS behaviors. Rare earth metal oxides as high-κ dielectrics are considered as the replacement of hafnium-based technology [17-19], among which Lu_2_O_3_ is the promising one as it shows well-insulating property, large bandgap (5.5 eV), better hygroscopic immunity, good thermal stability, and adequate dielectric constant of approximately 11 [20]. Gao et al. reported promising unipolar RS behavior in amorphous Lu_2_O_3_ oxide [21]. In contrast, we previously demonstrated the bipolar RS in various high-κ rare earth metal oxides, such as Tm_2_O_3_, Yb_2_O_3_, and Lu_2_O_3_, on silicon substrate [22]. The different RS behavior may be originated from their distinguished morphological changes. However, no flexible memory device has been demonstrated and detail switching dynamics is still unclear in this material. The superior experimental switching characteristics in Lu_2_O_3_ and room temperature deposition process allow it to be a possible functional material for flexible electronics. Therefore, in this study we investigate the RS behaviors of the sputter deposited lutetium sesquioxide (Lu_2_O_3_) thin film on flexible substrate for nonvolatile flexible memory application. In addition, we demonstrate that the memory performance of ReRAM on a flexible substrate has excellent electrical and mechanical reliabilities due to the high ductility of amorphous Lu_2_O_3_ thin film and the merit of the low-temperature process. Unlike other typical flexible resistive memory, better RS characteristics were achieved for advanced flexible memory applications.

## Methods

Flexible Ru/Lu_2_O_3_/ITO RS memory devices were fabricated on flexible polyethylene terephthalate (PET) substrates. The sputtered ITO-coated PET substrate was glued on a Si dummy wafer with polyimide tape to mechanically support the flexible substrate during fabrication process. The Lu_2_O_3_ thin films with a thickness of 20 nm were deposited by reactive radio frequency magnetron sputtering system in argon-oxygen (3:1) medium at room temperature from a Lu metal target. The chamber working pressure was maintained at 10 mTorr with the rf power of 130 W during deposition. The sputtering rate and time of the film were about 0.17 Å/s and 20 min, respectively. Finally, a 50-nm-thick square shape (100 × 100 μm^2^) Ru metal top electrode was deposited on the oxide films through shadow mask by DC sputtering technique operated at 10 mTorr in Ar environment.

The crystalline structure and the chemical compositions of the films were examined by x-ray diffraction (XRD) and x-ray photoelectron spectroscopy (XPS), respectively. The crystal structure of the Lu_2_O_3_/ITO film was determined in a Bruker-AXS D5005 diffractometer (Bruker Biosciences Inc., Billerica, MA, USA) using Cu K_α_ (*λ* = 1.542 Å) radiation. The composition and chemical bonding in the Lu_2_O_3_ film were analyzed using a Thermo Scientific Microlab 350 VG system (Thermo Fisher Scientific, Inc., Waltham, MA, USA) with a monochromatic Al K_α_ (1,486.7 eV) source. The surface of the Lu_2_O_3_ film was pre-sputtered using an Ar ion source. The chemical shifts in the spectra were corrected with reference to the C 1 *s* peak (from adventitious carbon) at a binding energy of 285 eV. Curve fitting was performed after Shirley background subtraction using a Lorentzian-Gaussian fitting. The roughness of the film was measured using an NT-MDT Solver P47 (NT-MDT Co., Zelenograd, Moscow, Russia). The atomic force microscope (AFM) was operated in the tapping mode for imaging. The electrical properties of the Ru/Lu_2_O_3_/ITO memory devices were measured by a semi-automated cascade measurement system equipped with Agilent E5260 high-speed semiconductor parameter analyzer (Agilent Technologies, Sta. Clara, CA, USA).

## Results and discussion

**Figure 1:**
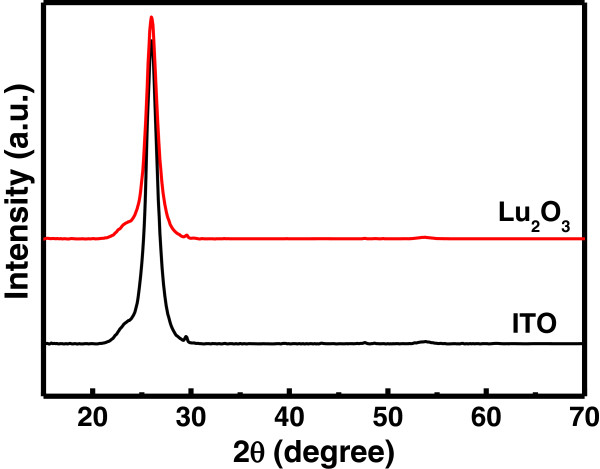
**XRD micrographs of amorphous Lu**_**2**_**O**_**3 **_**thin film sputtered on flexible ITO/PET substrate.**

**Figure 2:**
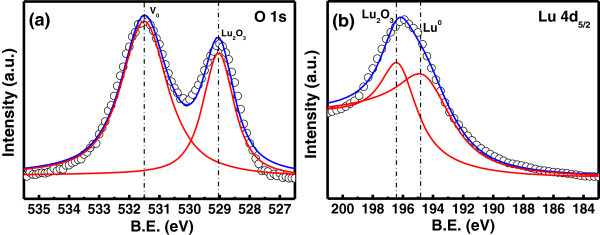
**XPS line-shape analyses. (a)** O 1 *s*. **(b)** Lu 4*d* spectra for Lu_2_O_3_ thin film on ITO/PET substrate.

**Figure 3:**
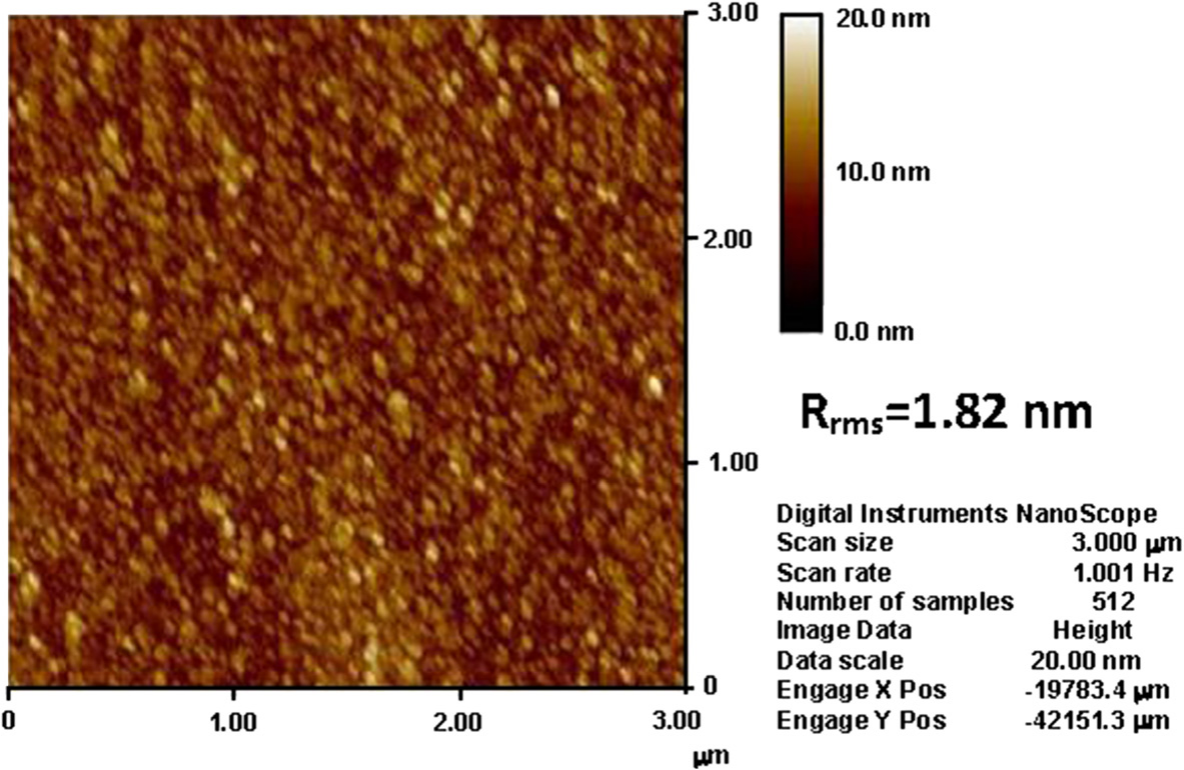
**AFM image of Lu**_**2**_**O**_**3 **_**thin film on flexible ITO/PET substrate.**

The grazing incident XRD spectra recorded on 20-nm thick as deposited Lu_2_O_3_ films on ITO/PET are shown in Figure 1. No diffraction peak was observed from the Lu_2_O_3_ film deposited at room temperature, which indicates that the films remain in amorphous phase. To investigate the compositional changes of the oxide, XPS analyses were performed on Lu_2_O_3_ thin films. Adventitious hydrocarbon C 1 *s* binding energy was used as a reference to correct the energy shift of O 1 *s* and Lu 4*d* core levels due to differential charging phenomena. The core levels of O 1 *s* and Lu 4*d* spectra with their appropriate peak curve-fitting lines for the Lu_2_O_3_ thin film are shown in Figure 2a,b, respectively. The O 1 *s* spectrum at the surface of Lu_2_O_3_ thin film consists of two binding energy peaks: a low binding energy peak at 529.2 eV for Lu_2_O_3_ and a high binding energy peak at 531.4 eV, usually attributed to oxide defects or nonlattice oxygen ions [23,24]. The Lu 4*d* line spectrum consists of a higher binding energy peak at 196 eV for Lu_2_O_3_ and a lower binding energy peak at 194.4 eV, which is attributed to the existence of Lu ions in the oxide thin film [23]. The presence of Lu ionic peak in Lu 4*d* line spectrum can be attributed to the formation of chemical defects, most probably the oxygen vacancies. Figure 3 shows the AFM images of 20-nm-thick Lu_2_O_3_ film. The rms value obtained by AFM observation was 1.82 nm. The lower surface roughness may result in better uniformity and higher yield of the fabricated memory devices.

**Figure 4:**
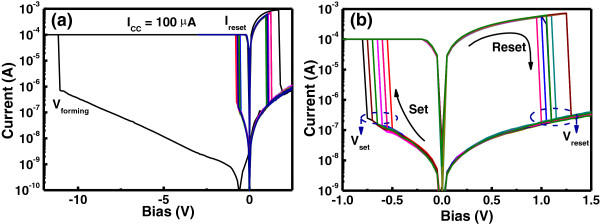
**Analysis of the RS characteristics of Ru/Lu**_**2**_**O**_**3**_**/ITO ReRAM device. (a)** The electroforming process of the Ru/Lu_2_O_3_/ITO ReRAM device with current compliance of 100 μA. Shaded area shows the typical RS behavior after electroforming process. **(b)** Enlarged view of the shaded region showing promising RS characteristics of the Ru/Lu_2_O_3_/ITO ReRAM device.

In order to investigate the memory performance of the flexible Ru/Lu_2_O_3_/ITO ReRAM cell, the RS characteristics were analyzed. A high bias voltage with predefined current compliance (*I*_CC_) of 100 μA was applied to the pristine memory cell to initiate the RS into the Lu_2_O_3_ thin film, as shown in Figure 4a. *I*_CC_ is required to protect the device from hard breakdown. During this initial bias sweeping, a sudden abrupt decrease in oxide conductance was observed, which is known as soft breakdown or electroforming process. A nanomorphological change into the oxide layer is assumed due to the introduction of a high oxygen vacancy density of the oxide thin films [25]. After the electroforming process, the memory device switches to low-resistance state (LRS). To change the resistance state of the memory device, a sufficient positive bias of certain value (*V*_reset_) was applied and the devices transform to high-resistance state (HRS), as shown in Figure 4b. In contrast, an application of negative bias results in a transition from HRS to LRS at certain set voltage (*V*_set_) and this effect is reproducible over several hundreds of voltage sweeping cycles. As can be seen that the Ru/Lu_2_O_3_/ITO ReRAM cell can be switched between two distinguished resistance state (HRS to LRS and *vice versa*), at a very low voltage of approximately 0.8 V (100 μA set current) and approximately 1.2 V (<1 mA reset current) for set and reset operations, respectively. The lower switching voltage is believed due to the low power hopping conduction via oxide defects [7]. In order to realize the current conduction mechanism into the Lu_2_O_3_ thin film, both HRS and LRS current–voltage (*I*-*V*) characteristics at different temperature were analyzed.

**Figure 5:**
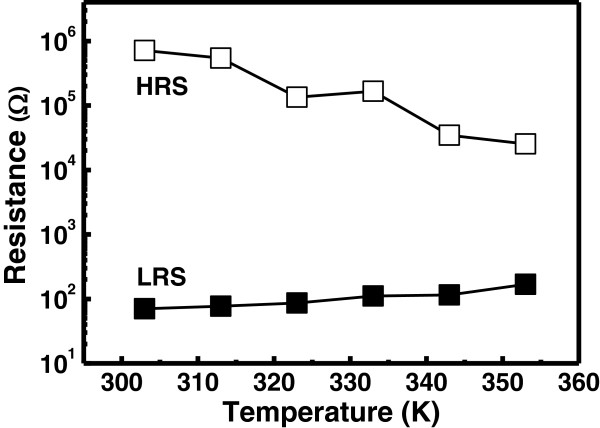
**Temperature-dependent resistance values of HRS and LRS in Ru/Lu**_**2**_**O**_**3**_**/ITO ReRAM device.**

**Figure 6:**
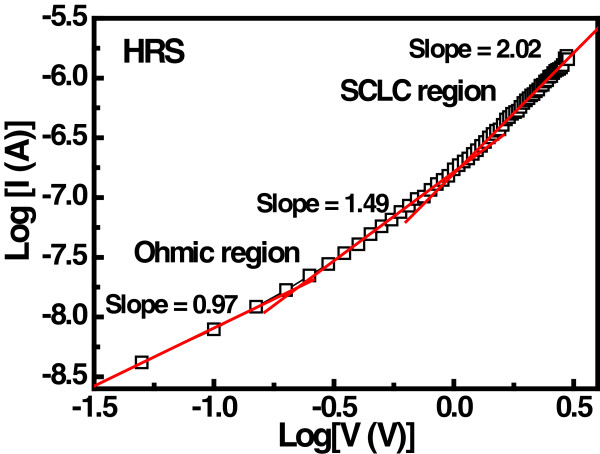
**Log( *I *) vs. log ( *V *) plot of Lu**_**2**_**O**_**3 **_**thin film at room temperature for SCLC conduction.**

Figure 5 shows the resistance variation of the memory device at different resistance states at different temperatures ranging from 303 to 353 K. In HRS, the resistance value decreases as the temperature increase to 353 K. These results can be associated with a semiconducting/oxide-like behavior of the oxide film. On the other hand, the LRS increases with increasing the temperature, indicating the formation of a metallic-like filament by percolation of oxygen vacancies and other ionic and electronic defects within or near the interface area [26]. Therefore, oxide defects mainly oxygen-vacancies-mediated filament conduction is believed to influence the RS behavior in the Ru/Lu_2_O_3_/ITO ReRAM device. The current conduction behavior at HRS and LRS is further analyzed. The double-logarithmic plot of room temperature *I-V* data at HRS for Lu_2_O_3_ thin film shows ohmic (*I* ∞ *V*) and quadratic (*I* ∞ *V*^2^) in Figure 6. Therefore, space-charge-limited-current (SCLC) conduction is dominant in Lu_2_O_3_ thin dielectric. For a single trap level, the SCLC conduction mechanism can be explained as follows [27,28]:

(1) $$I_{I}\;\infty \;qn_{0}\;\mu \frac{V}{t_{\mathrm{ox}}}$$

(2) $$I_{\mathrm{II}}\;\infty \;\frac{9}{8}\;\mu \epsilon_{0}\;\epsilon_{r}\frac{V^{2}}{{t_{\mathrm{ox}}}^{3}}$$

where *q* is an electronic charge, *n*_0_ is the effective free carrier density of traps in thermal equilibrium, *μ* is the electronic mobility of oxide, *t*_ox_ is the oxide thickness, *V* is the externally applied voltage, *ϵ*_*0*_ is the permittivity of free space, and *ϵ*_*r*_ is the dynamic dielectric. For an applied voltage across the oxide below 1.0 V, the slope of the log*I-*log*V* characteristic is on the order of 1.0 to approximately 2.0, which implies ohmic conduction, because the numbers of the injected electrons are lower compared to the thermally generated free electrons density (*n*_0_) inside the oxide film. When the applied voltage is higher than 1.0 V, the slopes are larger (≥2), which implies SCLC conduction. A transition from ohmic to SCLC region is observed when the injected carrier density exceeds the volume-generated free carrier density. The SCLC transition voltage can be expressed as follows [27,28]:

(3) $$V_{\mathrm{tr}}=\frac{8qn_{0}{t_{\mathrm{ox}}}^{2}}{9\epsilon_{0}\epsilon_{r}\theta }$$

(4) $$\theta =\frac{N_{C}}{g_{n}N_{t}}\exp\left(\frac{E_{t}-E_{C}}{k_{B}T}\right)$$

where *θ* is the ratio of free to total carrier density, *N*_c_ is the density of state in the conduction band, *g*_*n*_ is the degeneracy of the energy state in the conduction band, *N*_t_ is the trap density, *k*_*B*_ is the Boltzmann constant, and *E*_t_ and *E*_c_ are the trap and conduction band energy level, respectively. By further increasing the applied voltage, more carriers will be injected from the injecting electrode and a space charge region appears near the injecting electrode interface so that the injected excess carriers dominate the thermally generated charge carrier and hence the current increases rapidly.

**Figure 7:**
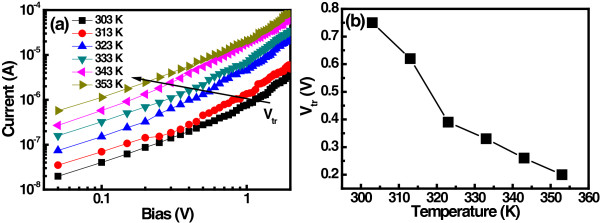
**Logarithmic plots of *I*-*V *characteristics in Lu**_**2**_**O**_**3 **_**thin film at different temperatures. (a)** Double-logarithmic plot of *I*-*V* characteristics in Lu_2_O_3_ thin film at 303 to 353 K temperature range. **(b)** Temperature-dependent *V*_tr_ plot of Lu_2_O_3_ thin film.

**Figure 8:**
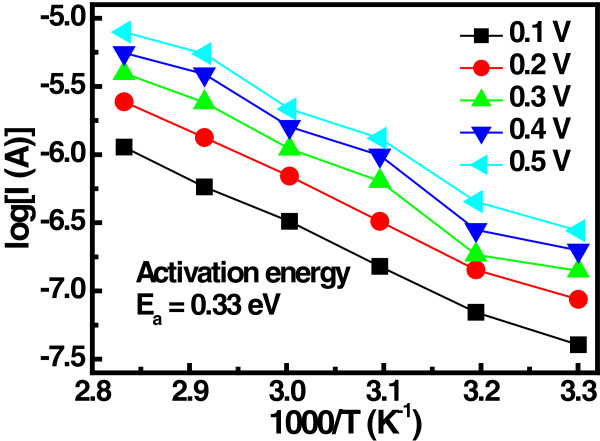
**Arrhenius plot for Lu**_**2**_**O**_**3 **_**ReRAM current conduction.** Activation energy obtained from the slope of the log(*I*) vs. 1/*T* curves.

**Figure 9:**
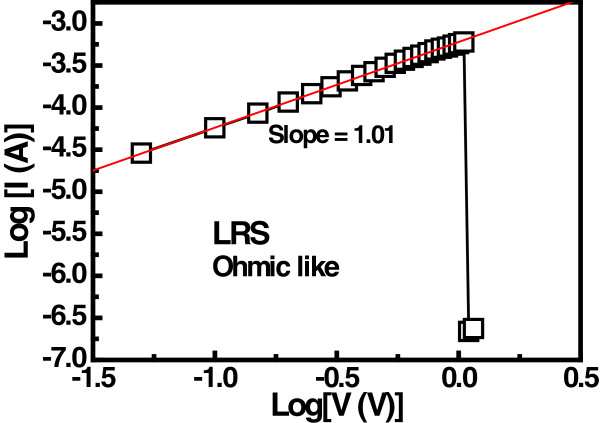
**Double-logarithmic plot of *I*-*V *** **characteristics of Ru/Lu**_**2**_**O**_**3**_**/ITO ReRAM device at LRS.**

Figure 7a shows the *I*-*V* characteristics of Lu_2_O_3_ thin film at different temperatures. The measured transition voltage (*V*_tr_) obtained from the *I*-*V* characteristics is depicted in Figure 7(b). It can be seen that the *V*_tr_ decreases with increasing temperature, suggesting that the thermal generation of the carrier increases with temperature. Relatively lower voltage is required to fill all the trap levels at higher temperature and hence *V*_tr_ decreases. The trapping level in the Lu_2_O_3_ thin film can be determined from the Arrhenius plot of the current for different electric fields. The activation energy (*E*_a_) was calculated from the slope ln(*I*)-1/*T* plot to be about 0.33 eV, as shown in Figure 8. The LRS I-V curves of Lu_2_O_3_ ReRAM devices were plotted on the logarithmic scale, as shown in Figure 9. The linear behavior of *I*-*V* curve of the ReRAM devices with a nearly constant slope value of approximately 1.01 suggests that the ohmic conduction is dominant in LRS conduction. This may be due to stochastic filament formation by accumulated oxide defects/vacancies into the Lu_2_O_3_ film [29].

**Figure 10:**
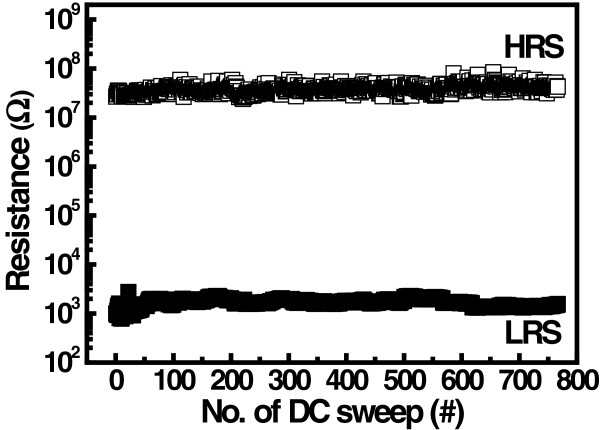
**Endurance characteristics of Ru/Lu**_**2**_**O**_**3**_**/ITO ReRAM device for continuous voltage sweeping operation.**

**Figure 11:**
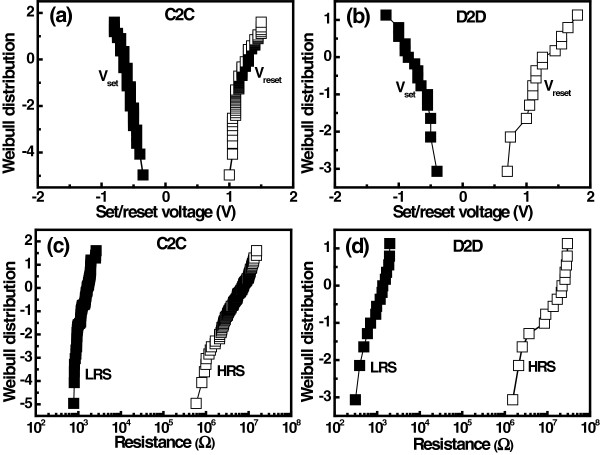
**Distributions of voltages for cycle-to-cycle and device-to-device measurements.** Weibull distribution of set/reset voltages for **(a)** C2C and **(b)** D2D measurements. HRS and LRS distributions of the device for **(c)** C2C and **(d)** D2D measurements.

Figure 10 depicts the memory switching characteristics for successive switching cycle. The resistance ratio between two memory states in Ru/Lu_2_O_3_/ITO ReRAM cell is maintained more than 10^3^ during the continuous memory switching, which is useful for NVM applications. Additionally, a good uniformity in resistance values at HRS and LRS was observed. This may be due to the smoother surface roughness of the Lu_2_O_3_ film. Good switching and device uniformity in memory device are an important factor for flexible ReRAM devices. Very few literatures have been reported on cycle-to-cycle (C2C) distribution (switching uniformity) of flexible NVM applications [10,30-32]. However, device-to-device (D2D) distribution (device uniformity) among different devices is very crucial for successful implementation of NVM technology. Figure 11 shows the Weibull distribution of switching voltages and resistance values of the Ru/Lu_2_O_3_/ITO ReRAM device. Randomly selected 15 ReRAM cells were measured for 100 switching cycle of each device. A very small dispersion was observed in both parameters as shown.

**Figure 12:**
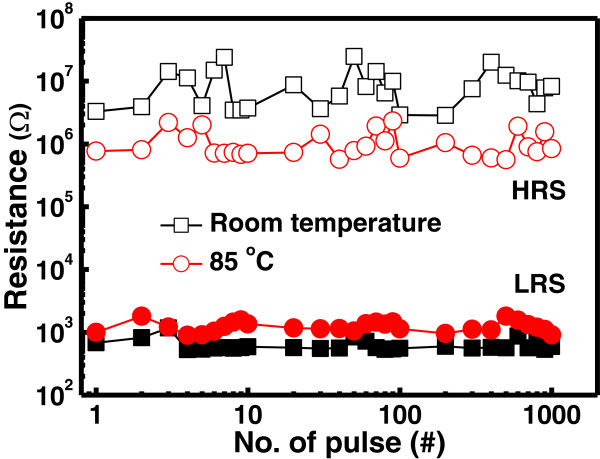
**Pulse switching endurance characteristics of Ru/Lu**_**2**_**O**_**3**_**/ITO ReRAM device at room temperature and 85°C.**

**Figure 13:**
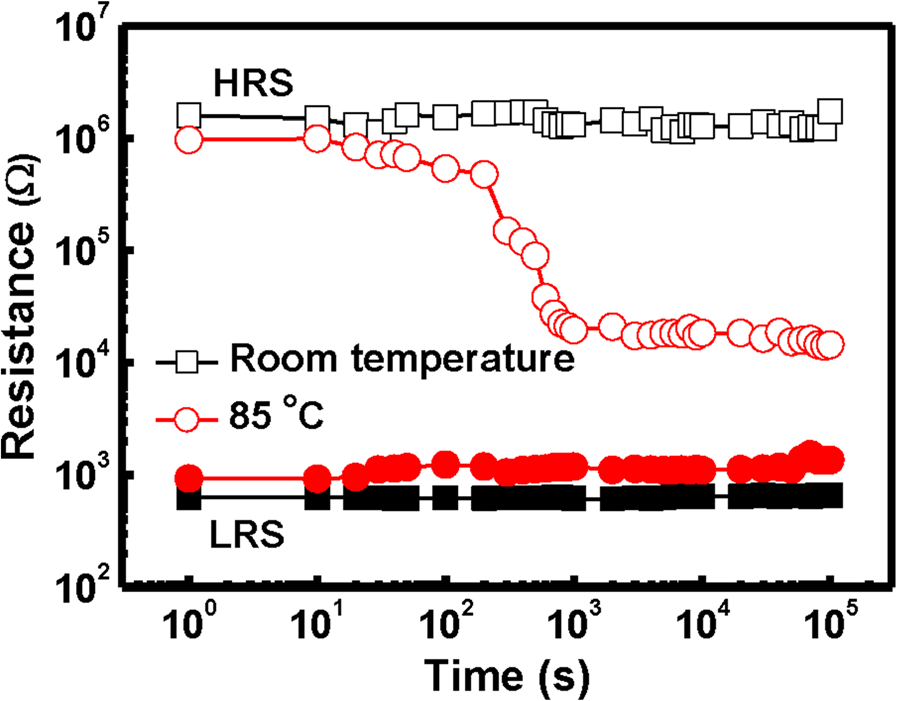
**Data retention characteristics of Ru/Lu**_**2**_**O**_**3**_**/ITO ReRAM device at room temperature and 85°C.**

**Figure 14:**
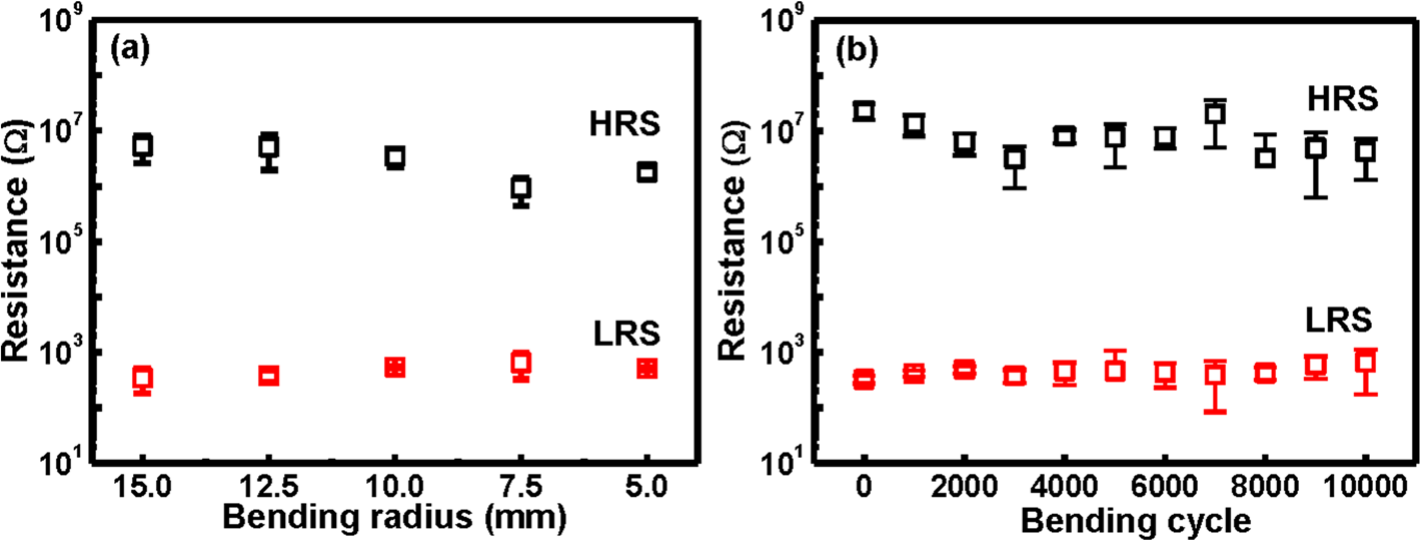
**Measurements of the flexibility and mechanical endurance of Ru/Lu**_**2**_**O**_**3**_**/ITO ReRAM device at various conditions. (a)** Flexibility test of Ru/Lu_2_O_3_/ITO ReRAM device for various bending curvature. **(b)** Mechanical bending endurance of Ru/Lu_2_O_3_/ITO ReRAM device at bending radius of 10 mm.

To understand the potentiality of Ru/Lu_2_O_3_/ITO flexible memory device, the reliability characteristics of impulse switching endurance, data retention, and mechanical endurance were characterized. Figure 12 shows the pulse switching endurance characteristics of the flexible memory device under ±2 V of impulse voltages, measured at room temperature and 85°C. After each pulse switching a reading voltage pulse of 0.1 V was applied for reading operation. The Ru/Lu_2_O_3_/ITO flexible memory device can be switched over 10^3^ program/erase (P/E) cycle maintaining a memory window of approximately 10^3^ at both room temperature and 85°C. Figure 13 shows the data retention characteristics of the Lu_2_O_3_ flexible memory devices after cyclic measurement at both room temperature and 85°C. Good data retention of 10^5^ s is obtained. A small fluctuation is observed at elevated temperature for endurance and retention test. This may be attributed to the generation and redistribution of oxide defects in the switching material [7,33] due to increase stress and temperature. In retention characteristics, a degradation behavior in memory window was observed, though a well resolved memory window of approximately 10 after 10^5^ s is maintained. This can be explained by the stress-induced leakage current via generated defects in the oxide thin films [7]. The flexibility and mechanical endurance are the key parameter for flexible electronic applications. The flexibility and mechanical endurance were also experienced for Ru/Lu_2_O_3_/ITO memory devices, as shown in Figure 14a,b, respectively. It was observed that good flexibility and mechanical endurance can be achieved in both devices. This may be due to the high ductility of thin Ru metal electrodes and the amorphous Lu_2_O_3_ oxide film in ReRAM structure. In addition, good mechanical endurance is also achieved up to 10^4^ of the bending cycle. The mechanical stress is applied by bending the Ru/Lu_2_O_3_/ITO flexible ReRAM device to a small 10-mm radius at every second, and the resistances were measure after each 1,000 bending cycle. As shown in Figure 14b, the device reveals a well-resolved memory window of approximately 10^2^ after 10^4^ of continuous bending cycle, indicating good flexibility of the Ru/Lu_2_O_3_/ITO ReRAM cell. The superior switching characteristics of the Ru/Lu_2_O_3_/ITO flexible ReRAM device show the potential for future flexible low-power electronic applications.

## Conclusions

In this work, the RS behavior in the Lu_2_O_3_ thin films on flexible PET substrate is explored for advanced flexible nonvolatile random access memory applications. The current conduction mechanism is dominated by the bulk-limited SCLC conduction in HRS and the ohmic-like conduction in LRS. A shallow trap level at 0.33 eV below the conduction band was evaluated in Lu_2_O_3_ thin films. The filament conduction via oxide defects was described for the RS behavior in the Lu_2_O_3_ thin films on ITO/PET substrate. Low-voltage RS and good device uniformity were obtained in the Ru/Lu_2_O_3_/ITO flexible ReRAM cell. Good memory reliability characteristics of switching endurance, data retention, flexibility, and mechanical endurance were promising for future memory applications. The superior switching behaviors in Ru/Lu_2_O_3_/ITO flexible ReRAM device have great potential for future advanced nonvolatile flexible memory applications.

## Competing interests

The authors declare that they have no competing interests.

## Authors’ contributions

SM designed the experiment, measured the data of the Ru/Lu_2_O_3_/ITO flexible ReRAM cell, and drafted the manuscript. JLH and KK provided useful suggestions and helped analyze the characterization results. TMP supervised the work and finalized the manuscript. All authors read and approved the final manuscript.
